# Fungal strain and crop cultivar affect growth of sweet pepper plants after root inoculation with entomopathogenic fungi

**DOI:** 10.3389/fpls.2023.1196765

**Published:** 2023-06-05

**Authors:** Liesbet Wilberts, Nicolas Rojas-Preciado, Hans Jacquemyn, Bart Lievens

**Affiliations:** ^1^ Centre of Microbial and Plant Genetics (CMPG) Laboratory for Process Microbial Ecology and Bioinspirational Management (PME&BIM), Department of Microbial and Molecular Systems (M2S) KU Leuven, Leuven, Belgium; ^2^ Leuven Plant Institute (LPI), KU Leuven, B-3001 Leuven, Belgium; ^3^ Laboratory of Plant Conservation and Population Biology, Biology Department, KU Leuven, Leuven, Belgium

**Keywords:** *Akanthomyces muscarius*, *Beauveria bassiana*, *Cordyceps fumosorosea*, endophyte, plant growth promotion

## Abstract

As endophytes, entomopathogenic fungi can protect plants against biotic and abiotic stresses and at the same time promote plant growth and plant health. To date, most studies have investigated whether *Beauveria bassiana* can enhance plant growth and plant health, while only little is known about other entomopathogenic fungi. In this study, we evaluated whether root inoculation of the entomopathogenic fungi *Akanthomyces muscarius* ARSEF 5128, *B. bassiana* ARSEF 3097 and *Cordyceps fumosorosea* ARSEF 3682 can promote plant growth of sweet pepper (*Capsicum annuum* L.), and whether effects are cultivar-dependent. Plant height, stem diameter, number of leaves, canopy area, and plant weight were assessed four weeks following inoculation in two independent experiments using two cultivars of sweet pepper (cv. ‘IDS RZ F1’ and cv. ‘Maduro’). Results showed that the three entomopathogenic fungi were able to enhance plant growth, particularly canopy area and plant weight. Further, results showed that effects significantly depended on cultivar and fungal strain, with the strongest fungal effects obtained for cv. ‘IDS RZ F1’, especially when inoculated with *C. fumosorosea*. We conclude that inoculation of sweet pepper roots with entomopathogenic fungi can stimulate plant growth, but effects depend on fungal strain and crop cultivar.

## 1 Introduction

Entomopathogenic fungi are well known for their ability to infect and kill insects (Shah and Pell, 2003; Islam et al., 2021). After invading a host, the fungus proliferates and invades the host’s organs and tissues, leading to the death of the insect. Next, the fungus emerges from the insect cadaver and produces thousands of new spores, which then disperse and infect a new host (Shah and Pell, 2003; Islam et al., 2021). Due to the fact that they are able to suppress natural insect populations and generally impose no or minimal adverse effects on humans and the environment (but see Hu et al., 2016), entomopathogenic fungi are commonly used as bioinsecticides, especially because virtually all insect orders are vulnerable to fungal diseases (Hajek and St Leger, 1994; Glare et al., 2012; Bamisile et al., 2021). There are several products based on entomopathogenic fungi commercially available for insect control, predominantly based on members of the genera *Akanthomyces* (previously *Lecanicillium* and *Verticillium*) (Hypocreales: Cordycipitaceae), *Beauveria* (Hypocreales: Cordycipitaceae), *Cordyceps* (previously *Isaria* and *Paecilomyces*) (Hypocreales: Cordycipitaceae) and *Metarhizium* (Hypocreales: Clavicipitaceae) (Faria and Wraight, 2007; van Lenteren et al., 2018).

In addition to colonizing insect hosts as pathogens, an increasing number of studies have shown that entomopathogenic fungi can associate with plants, often by colonizing plant tissues without causing disease symptoms as endophytes (Vega, 2008; Vidal and Jaber, 2015; Gange et al., 2019; Quesada-Moraga, 2020). Local or systematic colonization occurs mainly in the roots, stems, leaves and internal tissues of plants (Behie et al., 2015). The endophytic behavior of entomopathogenic fungi has been reported in numerous cultivated and non-cultivated plant species, both naturally colonized and artificially inoculated by diverse methods, and several of these fungi have the potential to improve the plant’s response to biotic and abiotic stresses (Vega, 2008; Vidal and Jaber, 2015; Vega, 2018; Gange et al., 2019; Francis et al., 2022). For example, banana and common bean plants inoculated with entomopathogenic fungi showed reduced reproduction rates and higher mortality rates of the banana root borer (*Cosmopolites sordidus*), one of the most important pests on bananas (Akello et al., 2008), and the pea leaf miner (*Liriomyza huidobrensis*) (Akutse et al., 2013), respectively, while endophytic colonization of sweet pepper by entomopathogenic fungi had negative effects on the development and fecundity of aphids (*Myzus persicae*) (Jaber and Araj, 2018; Wilberts et al., 2022). Moreover, endophytic entomopathogenic fungi have been shown to reduce pathogen infestation (Jaber and Alananbeh, 2018; Jaber and Ownley, 2018) and provide plants with drought stress tolerance (Ferus et al., 2019).

Given their capability to increase plant resistance against biotic and abiotic stress, endophytic entomopathogenic fungi are being increasingly evaluated as biostimulants or biopesticides (Lacey et al., 2015; Lugtenberg et al., 2016; Jaber and Ownley, 2018; Vega, 2018; Quesada-Moraga, 2020). However, most studies exploring the potential of endophytic entomopathogenic fungi in agricultural sustainability have focused on their use as biocontrol agents to suppress insect pests (Vidal and Jaber, 2015; Vega, 2018; Mantzoukas and Eliopoulos, 2020) and less research has focused on their possible role as plant growth promoters, notwithstanding a number of studies have shown their potential to stimulate plant growth following endophytic colonization (Tall and Meyling, 2018; Canassa et al., 2019; Espinoza et al., 2019; Ahmad et al., 2020). Given that endophytic entomopathogenic fungi can persist for a long time in host tissues, growth-promoting effects can be expected to last for a long time (Brownbridge et al., 2012; Bamisile et al., 2020), although there are also examples of transient colonization that led to enhanced growth (Gurulingappa et al., 2010; Resquín-Romero et al., 2016), further enhancing their appeal as plant growth promoters.

Among endophytic fungal entomopathogens, *Beauveria bassiana* is the most frequently studied species to promote plant growth (Vega, 2018). It has been reported as early as 1990 as naturally occurring in maize (Vakili, 1990), and has since then been isolated from several other plant species (Márquez et al., 2007; Vega et al., 2010; Pimentel et al., 2016). The fungus has also been successfully established as an endophyte in several crops following artificial inoculation, benefiting plant growth and overall plant health (Espinoza et al., 2019; Saragih et al., 2019; Shaalan et al., 2021). By contrast, only little attention has been given to other fungal entomopathogens like *Akanthomyces* or *Cordyceps*, and their potential benefits on plant growth and plant health remain to be investigated. Furthermore, the effects of entomopathogenic fungi have been shown to vary between plant species (Gurulingappa et al., 2010; Sánchez-Rodríguez et al., 2018), suggesting that plant growth promotion may be affected by the host’s genotype or cultivar. Because plant-fungus interactions comprise complex molecular dialogues that induce large-scale transcriptomic changes in both partners (Tucci et al., 2011; Pieterse et al., 2014; Alam et al., 2021; Mattoo and Nonzom, 2021), it can be assumed that both the entomopathogenic fungal strain and cultivar strongly determine the net result of the plant response, but evidence is still scarce.

The aim of this study was to assess the plant growth promoting capabilities of different species of entomopathogenic fungi and to assess whether plant responses are mediated by plant cultivar. Therefore, we tested the effects of root inoculation of two cultivars of sweet pepper (*Capsicum annuum* L.; Solanaceae) with *B. bassiana* (ARSEF 3097) and the fungal species *Akanthomyces muscarius* (ARSEF 5128) and *Cordyceps fumosorosea* (ARSEF 3682) on plant height, stem diameter, number of leaves, canopy area and plant weight. Experiments were performed in two different years.

## 2 Materials and methods

### 2.1 Plant and fungal material

Two cultivars of sweet pepper were used in this study: cv ‘IDS RZ F1’ (Rijk Zwaan, De Lier, the Netherlands) and cv ‘Maduro’ (Enza Zaden, Enkhuizen, the Netherlands). These cultivars are commonly used in commercial sweet pepper cultivation in Belgium. Both cultivars have crude, medium-size red fruits. IDS RZ F1 is resistant to Tobamovirus pathotypes P0, P1, P2 and P3, while Maduro is resistant to pathotypes P0, P1 and P2. Plants were sown in a 3:1 mixture of potting mix (Universal potting mix; Agrofino, Ghent, Belgium) and white sand, and incubated until fungal inoculation (see further) in a plant cabinet that was equipped with LED lights above the foliage, providing a photosynthetic flux density of 790 µmol photons m^-2^s^-1^ (23 ± 1°C, 65 ± 2% RH and a 16L:8D photoperiod) (MD1400, Snijders Labs, the Netherlands). Three endophytic entomopathogenic fungi were used in this study: *Akanthomyces muscarius* ARSEF 5128 (Ve-6; previously known as *Lecanicillium muscarium*), *Beauveria bassiana* ARSEF 3097 (ATCC 74040), and *Cordyceps fumosorosea* ARSEF 3682 (Apopka 97; previously identified as *Isaria fumosorosea*). These three fungi are the active substance in the bioinsecticides Mycotal^®^, Naturalis^®^ and PreFeRal^®^, respectively. Originally, *A. muscarius* ARSEF 5128 was isolated from a greenhouse whitefly in Littlehampton (UK) (Hall, 1982), *B. bassiana* ARSEF 3097 from a boll weevil in the Rio Grande Valley (USA) (Wright, 1996) and *C. fumosorosea* ARSEF 3682 from a mealy bug in a greenhouse in Apopka (USA) (Vidal et al., 1998). All strains have been shown to colonize plants as an endophyte upon artificial inoculation in various crops, including sweet pepper (Kuchár et al., 2019; Rondot and Reineke, 2019; Nicoletti and Becchimanzi, 2020; Doherty et al., 2021; Wilberts et al., 2022). The strains were acquired from the Agricultural Research Service Collection of Entomopathogenic Fungal Cultures (ARSEF; New York, USA), and were stored as agar plugs in glycerol at -80°C.

### 2.2 Fungal spore suspensions and plant inoculation

Stored agar plugs of each fungus were plated onto quarter-strength (¼) Sabouraud dextrose agar with yeast extract (Oxoid Holdings Ltd, United Kingdom) (SDAY), and once again replated onto the same agar medium before use. Conidial suspensions were prepared by culturing the fungi in darkness on SDAY for seven days at 25°C, followed by flooding the plates with sterile physiological water (0.8% NaCl) and scraping fungal tissue of the plates. Next, fungal fragments and spores were filtered through microcloth (Mira Cloth, Merck, Massachusetts, USA) to remove fungal hyphae, and the spore concentration was determined by using a Bürker hemocytometer under the microscope, and diluted to 1×10^7^ conidia mL^-1^. Before inoculation, a 100 µL aliquot of 1×10^3^ spores mL^-1^ was plated on three SDAY plates to check spore viability. The number of germinated and ungerminated spores was determined under the microscope after 24 h of incubation at 25°C. Spores with germ tubes at least two times longer than their diameter were considered as germinated. The germination assays showed >90% viability rate for all fungal spore suspensions used in the experiments.

Plants were inoculated as described in Wilberts et al. (2022). Briefly, at the first true leaf stage seedlings were uprooted and roots were rinsed under running tap water. Next, roots were dipped in 10 mL of the conidial spore suspensions for 18h. Roots of a separate set of seedlings were submerged in 10 mL physiological water to be included as non-inoculated (control) plants. Seedlings were then placed individually in 17-cm-diameter plastic pots in a 3:1 mixture of potting mix (Universal potting mix; Agrofino, Ghent, Belgium) and white sand (for chemical characteristics of the potting medium, see **Table S1**; Supporting information), and put in the greenhouse according to a randomized complete block design. The experiment was performed with 10 replicates per treatment, yielding 2 cultivars × 4 treatments × 10 plants = 80 plants in total. The experiment was performed twice (February-March 2021 and February-March 2022, further referred to as “Exp 2021” and “Exp 2022”, respectively). In both trials, plants were maintained at 23 ± 5°C, 65 ± 10% RH and a photoperiod of 16L:8D. Plants were watered daily with a nutrient solution for sweet pepper (**Table S2**; Supporting information). Temperature, relative humidity and solar insolation in the greenhouse were monitored throughout the experiments (**Figure S1**; Supporting information).

### 2.3 Plant growth

To assess plant growth, plant height (from lowest leaf node to the highest node), stem diameter, number of leaves, canopy area, and fresh and dry weight were measured for each plant. Plant height was measured at the start of the experiment (i.e. immediately after inoculation and potting) and subsequently every week for a total period of four weeks. All other variables were measured at the end of the experiment, i.e. four weeks after transplantation. Stem diameter was measured 1 cm above the lowest leaf node with a sliding caliper. Canopy area was calculated from top view images taken with a Canon EOS 1300d camera with Canon zoom lens EF-S 18-55mm f/3.5-5.6 III. The surroundings of the plants, including the plant pots, were covered with blue plastic as a contrast, while a red reference card of known size (15 × 10 cm) was put next to each plant. Then, canopy area was calculated by color segmentation with an R tool based on the EBImage (Pau et al., 2010) and imagemagick packages by separating the green plant pixels from the blue background. The red reference surface was used to calculate the green area (van Wesemael et al., 2019). To determine fresh and dry weight of the plants, plants were removed from the pots and roots were washed. Next, after air drying, fresh weight of the plants was determined. Subsequently, the plants were placed in individual paper bags and dried for five days at 80°C, after which the dry weight was determined. Before weighing the plants, the fifth leaf of every plant was collected, surface-sterilized (Landa et al., 2013) and subjected to DNA extraction and PCR amplification using the species-specific primer combinations ITS1F (Gardes and Bruns, 1993) and Am_Rv1 (5’-AGATGCTGATAATACAGAGTT-3’), ITS1F and Bb_Rv1 (5’-GATGCTGGAATACAAGAGTTTGAG-3’) and ITS1F and Cf_Rv1 (5’-CGGATTCAGAAAGACTGATAG-3’) to detect *A. muscarius*, *B. bassiana* and *C. fumosorosea* respectively, as described in Wilberts et al. (2022).

### 2.4 Statistical analysis

Plant height was analyzed using a Generalized linear mixed model (GLMM) based on a Gamma distribution with a log link function using treatment, plant cultivar, and week as fixed factors, while plant was entered as random factor (performed with the ‘glmer’ function from the lme4 package). Plant height was entered as response variable, and the interaction factor between the fungal treatment and cultivar was added to the model. Stem diameter, canopy area, fresh weight and dry weight were analyzed using a Generalized Linear Model (GLM) based on a Gamma distribution with a log link function using treatment, plant cultivar and their interaction as fixed factors (performed with the ‘glm’ function from the lme4 package). The number of leaves was analyzed using a GLM based on a Poisson distribution with a log link function using treatment, plant cultivar and their interaction as fixed factors. For this analysis, each plant was considered a biological replicate, giving a total of 10 replicates per treatment. To evaluate overall differences between the different treatments and cultivars, an analysis of variance (ANOVA) Type III test was performed on all models. When an overall difference was observed, a *post hoc* pairwise comparison (with estimated marginal means using the emmeans package) was performed to determine the pairwise differences between the different treatments and cultivars. The statistical analysis of the greenhouse experiments was performed for each dataset separately, as experiments were performed in different years. A significance level of α = 0.05 was applied to establish significant differences. All analyses and visualization of the data (ggplot2 package) were performed using R version 3.6.1 (R Core Team, 2019).

## 3 Results

### 3.1 Plant growth

**Figure 1:**
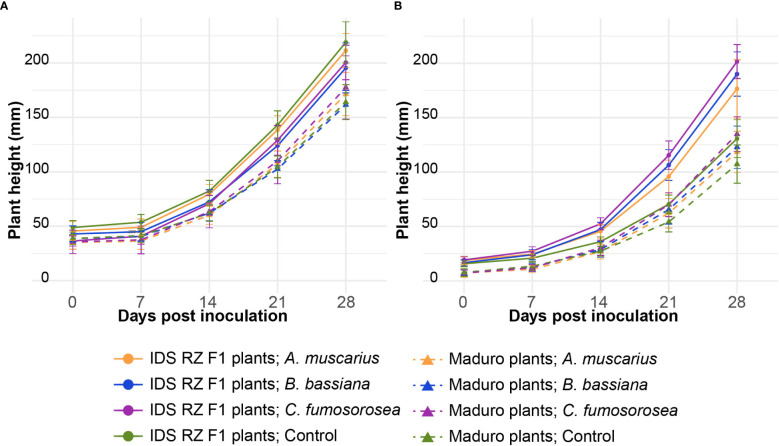
Average plant height of *Capsicum annuum* cv. IDS RZ F1 and cv. Maduro, inoculated with *Akanthomyces muscarius* ARSEF 5128, *Beauveria bassiana* ARSEF 3097 or *Cordyceps fumosorosea* ARSEF 3682, compared to control plants (*n* = 10). Plant height was measured weekly over a period of four weeks after fungal inoculation. The experiment was set up twice: in February-March 2021 **(A)** and in February-March 2022 **(B)**. Error bars represent standard error of the mean.

Cultivar had a strong effect on plant growth, while the effects of fungal strain were less pronounced and differed between the two experiments (**Table 1**). The effect of fungal strain on plant growth was strongest in the experiment performed in 2022 (**Table 1**). Plant height of IDS RZ F1 plants was significantly larger than that of Maduro plants over the course of both experiments (**Figure 1**; **Table 1**). In the experiment performed in 2021 (Exp 2021), fungal inoculation with the entomopathogenic fungi did not have a significant effect on plant height (**Table 1**). In the experiment performed in 2022 (Exp 2022), fungal inoculation resulted in higher IDS RZ F1 plants, especially when inoculated with *C. fumosorosea* (*P* = 0.019). For Maduro plants, fungal inoculation did not elicit an effect on plant height compared to control plants (*A. muscarius*: *P* = 0.997; *B. bassiana*: *P* = 0.967; *C. fumosorosea*: *P* = 0.868).

**Table 1:** Effects of fungal strain, cultivar and their interaction on growth of sweet pepper plants^1^.

		2021			2022	
	Fungal strain	Cultivar	Fungal strain × Cultivar	Fungal strain	Cultivar	Fungal strain × Cultivar
Plant height	5.372	9.269 **	2.426	8.945 *	32.321 ***	3.049
Stem diameter	8.115 *	1.594	7.315	10.362 *	5.802 *	1.02
Number of leaves	5.860	2.274	3.401	3.356	2.020	0.835
Canopy area	6.834	11.949 ***	3.233	54.902 ***	21.868 ***	4.847
Fresh weight	2.014	13.685 ***	14.314 **	34.132 ***	15.730 ***	0.560
Dry weight	5.704	14.426 ***	19.432 ***	39.289 ***	19.020 ***	1.947

^1^Chi-square distribution values from ANOVA on 10 plants per treatment measured four weeks after inoculation for all growth variables except plant height. Plant height was compared over the course of four weeks with weekly measurements (Generalized Linear Mixed Model). Asterisks indicate significant differences between the treatments (0.05 > P > 0.01: *; 0.01 > P > 0.001: **P < 0.001: ***).

**Figure 2:**
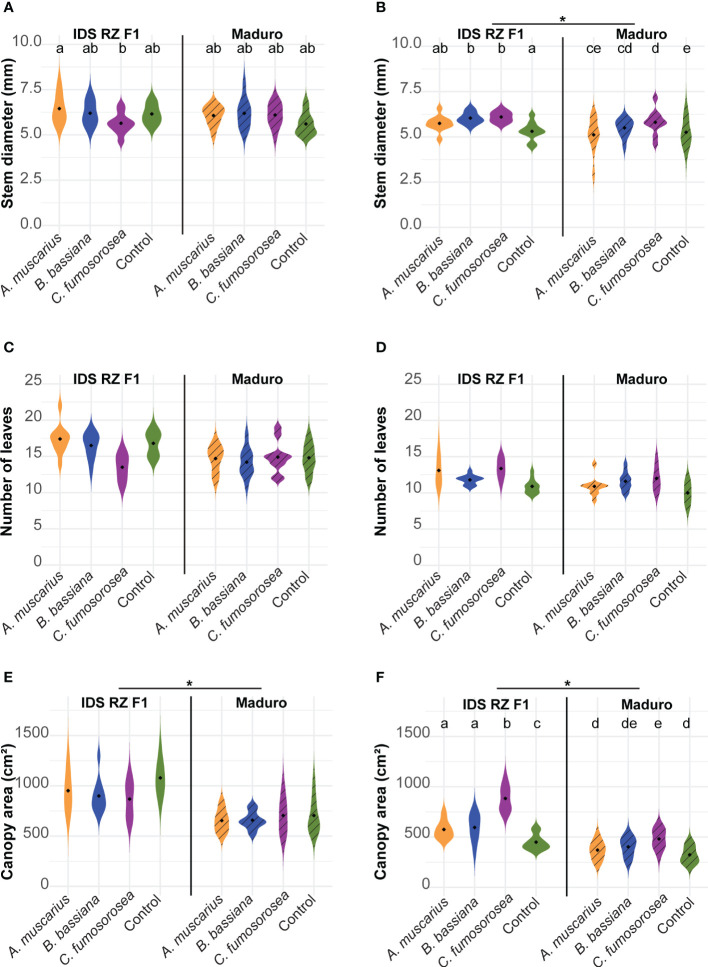
Stem diameter **(A, B)**, number of leaves **(C, D)**, canopy area **(E, F)** of *Capsicum annuum* cv. IDS RZ F1 and cv. Maduro, inoculated with *Akanthomyces muscarius* ARSEF 5128, *Beauveria bassiana* ARSEF 3097 or *Cordyceps fumosorosea* ARSEF 3682 compared to control plants four weeks after fungal inoculation (*n* = 10). The experiment was set up twice: in February-March 2021 **(A, C, E)** and in February-March 2022 **(B, D, F)**. Asterisks indicate a significant difference between the two cultivars (ANOVA, *P* < 0.05). Different letters indicate significant differences between treatments (Generalized linear model, *P* < 0.05). When no letters are given, no significant differences were observed.

Stem diameter did not differ between cultivars in Exp 2021, while in Exp 2022 Maduro plants were thinner than IDS RZ F1 plants (**Figures 2A, B**; **Table 1**). In the first experiment, IDS RZ F1 plants inoculated with *A. muscarius* were significantly thicker than plants inoculated with *C. fumosorosea* (*P* = 0.028), while no other differences were observed among treatments (**Figure 2A**). In the second experiment, plants inoculated with *B. bassiana* and *C. fumosorosea* had significantly thicker stems than control plants for both cultivars (IDS RZ F1 - *B. bassiana*: *P* = 0.037; IDS RZ F1 - C*. fumosorosea*: *P* = 0.020; Maduro - *B. bassiana*: *P* = 0.034; Maduro - C*. fumosorosea*: *P* < 0.001) (**Figure 2B**). Likewise, Maduro plants inoculated with *C. fumosorosea* had significantly thicker stems than Maduro plants inoculated with *A. muscarius* (*P* = 0.042) (**Figure 2B**).

The number of leaves did not differ significantly between cultivars in both experiments (**Table 1**). Also fungal inoculation did not affect the number of leaves significantly (**Figures 2C, D**; **Table 1**). Canopy area of IDS RZ F1 plants was significantly larger than that of Maduro plants in both experiments (**Figures 2E, F**; **Table 1**). While fungal inoculation did not significantly affect canopy area in Exp 2021, clear effects were observed in Exp 2022 (**Figures 2E, F**). Specifically, in Exp 2022, fungal inoculation of IDS RZ F1 plants resulted in a wider canopy for all fungi compared to the control plants (*A. muscarius*: *P* = 0.043; *B. bassiana*: *P* = 0.015; *C. fumosorosea*: *P* < 0.001). Furthermore, IDS RZ F1 plants inoculated with *C. fumosorosea* had a significantly wider canopy than IDS RZ F1 plants inoculated with *A. muscarius* or *B. bassiana* (*A. muscarius*: *P* < 0.001; *B. bassiana*: *P* < 0.001) (**Figure 2F**). IDS RZ F1 control plants had a canopy area of 449.57 ± 72.50 cm² on average, compared to 574.98 ± 86.46 cm², 595.22 ± 129.37 cm² and 883.44 ± 116.90 cm² for IDS RZ F1 plants inoculated with *A. muscarius*, *B. bassiana* and *C. fumosorosea*, respectively. Maduro plants inoculated with *C. fumosorosea* also had a wider canopy than Maduro plants inoculated with *A. muscarius* (*P* = 0.026) and control plants (*P* < 0.001), although the difference was less pronounced than in IDS RZ F1 plants. Maduro plants inoculated with *C. fumosorosea* had a canopy area of 481.47 ± 94.04 cm² on average, while Maduro plants inoculated with *A. muscarius* and control plants had an average canopy area of 370.16 ± 86.91 cm² and 323.73 ± 79.04 cm², respectively. Maduro plants inoculated with *B. bassiana* had a canopy area of 402.38 ± 90.40 cm² on average (**Figure 2F**).

**Figure 3:**
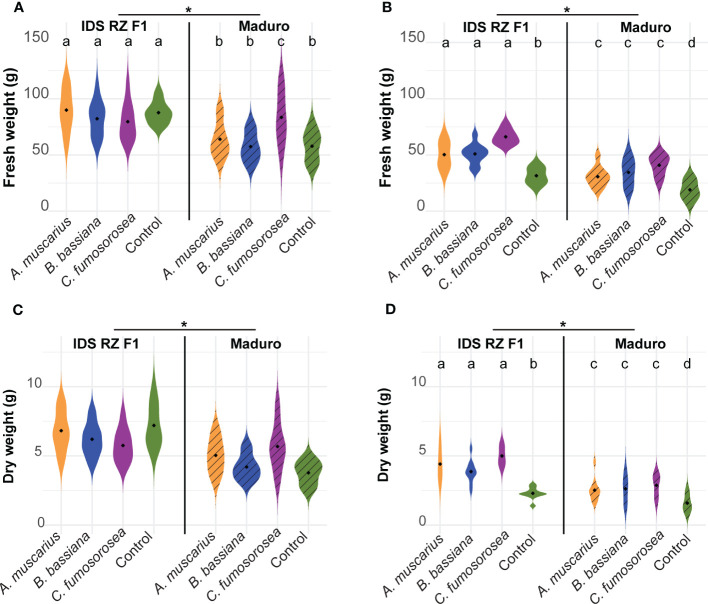
Fresh weight **(A, B)** and dry weight **(C, D)** of *Capsicum annuum* cv. IDS RZ F1 and cv. Maduro, inoculated with *Akanthomyces muscarius* ARSEF 5128, *Beauveria bassiana* ARSEF 3097 or *Cordyceps fumosorosea* ARSEF 3682 compared to control plants four weeks after fungal inoculation (*n* = 10). The experiment was set up twice: in February-March 2021 **(A, C)** and in February-March 2022 **(B, D)**. Asterisks indicate a significant difference between the two cultivars (ANOVA, *P* < 0.05). Different letters indicate significant differences between treatments (Generalized linear model, *P* < 0.05). When no letters are given, no significant differences were observed.

Fresh weight of IDS RZ F1 plants was higher than that of Maduro plants in both experiments (**Figures 3A, B**; **Table 1**). In Exp 2022, fresh weight of plants inoculated with the entomopathogenic fungi was significantly higher than that of control plants for both cultivars (IDS RZ F1 - A*. muscarius*: *P* = 0.001; IDS RZ F1 - *B. bassiana*: *P* < 0.001; IDS RZ F1 - C*. fumosorosea*: *P* < 0.001; Maduro - A*. muscarius*: *P* < 0.001; Maduro - *B. bassiana*: *P* < 0.001; Maduro - C*. fumosorosea*: *P* < 0.001) (**Figure 3B**). IDS RZ F1 plants inoculated with *A. muscarius*, *B. bassiana* and *C. fumosorosea* had a fresh weight of 50.28 ± 11.24 g, 50.96 ± 83.48 g and 66.18 ± 6.28 g on average, respectively, while IDS RZ F1 control plants weighted 31.56 ± 6.81 g on average. Maduro plants inoculated with *A. muscarius*, *B. bassiana* and *C. fumosorosea* weighted 30.69 ± 8.62 g, 34.51 ± 11.49 g and 40.90 ± 8.73 g on average, respectively, while Maduro control plants only weighted 18.98 ± 8.13 g (**Figure 3B**). Similarly, dry plant weight was significantly higher in inoculated plants compared to control plants (IDS RZ F1 - A*. muscarius*: *P* < 0.001; IDS RZ F1 - *B. bassiana*: *P* < 0.001; IDS RZ F1 - C*. fumosorosea*: *P* < 0.001; Maduro - A*. muscarius*: *P* = 0.002; Maduro - *B. bassiana*: *P* < 0.001; Maduro - C*. fumosorosea*: *P* < 0.001) (**Figure 3D**). In contrast to Exp 2022, an effect of fungal inoculation on plant weight was not observed in Exp 2021 (**Figure 3C**). However, both for fresh weight and dry weight, there was an interaction effect between cultivar and treatment in Exp 2021. This interaction effect was not observed in Exp 2022 (**Table 1**).

### 3.2 Endophytic colonization of the plants

At the end of both experiments, endophytic colonization by the three fungi was assessed by subjecting a sample from the fifth true leaf from all investigated plants to PCR analysis. The inoculated fungi could not be detected in any leaves of either inoculated or control plants four weeks after inoculation.

## 4 Discussion

In this study, we investigated the plant growth promoting capabilities of different species of entomopathogenic fungi and assessed whether plant responses were mediated by plant cultivar. Overall, entomopathogenic fungi had positive effects on plant growth parameters. However, effects were more pronounced in the experiment performed in 2022 compared to the experiment performed in 2021, possibly due to different climatic factors, although both experiments were set-up in the same way in the same period of the year (**Figure S1**, Supporting information). Similarly, previous studies have shown that entomopathogenic fungi like *B. bassiana* promote plant growth in diverse plant species, including chive (Espinoza et al., 2019), cucumber (Shaalan et al., 2021), bean (Jaber and Enkerli, 2016), grapevine (Mantzoukas et al., 2021), maize (Tall and Meyling, 2018; Liu et al., 2022), red chili (Saragih et al., 2019), and wheat (Guzmán et al., 2021). By contrast, there are also studies that found no or sometimes negative effects of endophytic entomopathogenic fungi on plant growth (Vega, 2018; Moloinyane and Nchu, 2019). Our results also showed that plant growth promoting effects differ with fungal species. Specifically, we found that inoculation with *C. fumosorosea* resulted in the strongest growth promotion of sweet pepper, while effects of inoculation with *A. muscarius* and *B. bassiana* were less pronounced.

Although most growth variables were affected by fungal inoculation in the 2022 experiment, fungal inoculation had the largest effect on leaf area and consequently plant weight. Plants inoculated with the tested entomopathogenic fungi had larger leaves and a larger canopy area, which can have strong implications for crop yield. With a greater canopy area, photosynthesis can be enhanced, vegetative growth increased, and consequently the aging of the plant delayed (Worku et al., 2007; Jo and Shin, 2020). Therefore, most studies on plant growth include leaf and/or canopy area as a major growth parameter, as plant weight is often too general as a parameter for plant development (Jo and Shin, 2020). It needs to be noted, however, that effects in our study were evaluated up to four weeks after fungal inoculation. While we specifically focused on vegetative growth in this study, further studies should be performed on how the observed growth promotion by fungal inoculation affects the growth of sweet pepper when the plants are balancing vegetative and generative growth.

Effects of fungal treatments resulted in similar trends in both cultivars. However, effects were more pronounced in the IDS RZ F1 cultivar, resulting in stronger significant differences between the treatments, while fungal treatments more often had a small to neutral effect on Maduro plant growth. Similarly, Canassa et al. (2020) found differences in plant growth between strawberry cultivars upon inoculation with entomopathogenic fungi. Fungal colonization of the internal parts of a plant is mediated by various biomolecules which drive dynamic changes in the expression of genes in the host plant and the fungus (Pieterse et al., 2014; Mattoo and Nonzom, 2021), and consequently can lead to strain- and cultivar-dependent differences. Furthermore, differences in plant colonization degree may affect plant responses (Jaber and Ownley, 2018). In our study, inoculated fungi could not be detected at the end of the experiment, suggesting that endophytic colonization was transient or that the fungi did not establish systematically in the plants, or at least not in the investigated leaf tissues (fifth leaf). Colonization of plant tissue by entomopathogenic fungi may be transient, with recovery of the fungi only in the first days after inoculation, especially when plants are grown in non-sterile soil, as was the case in this study (Posada et al., 2007; Gurulingappa et al., 2010; Allegrucci et al., 2017). Many factors may affect the degree to which entomopathogenic fungi colonize plant tissue, including inoculation method, environmental conditions and competing rhizosphere and endosphere microorganisms (Tefera and Vidal, 2009; Parsa et al., 2018; Rajab et al., 2020), but the exact mechanisms and forces behind endophytic colonization by entomopathogenic fungi still remain to be elucidated (Vega, 2018). Nevertheless, despite limited or even no endophytic colonization, beneficial effects of inoculation with entomopathogenic fungi have been observed, indicating that long term colonization or systemic colonization is not required to induce positive fungus-mediated effects (Parsa et al., 2018; Tall and Meyling, 2018). Further research should explore how and to which extent our plants were colonized by the fungal strains and how this affected plant responses. Regardless of fungal treatments, there were clear differences between both sweet pepper cultivars. In both experiments performed, Maduro plants were shorter, had smaller leaves and weighed significantly less than IDS RZ F1 plants. Contrary to our results, Maduro is described as generally slightly bigger than IDS RZ F1 according to the cultivar description. On the other hand, IDS RZ F1 is selected to produce fruits somewhat earlier than Maduro, so it is possible that young IDS RZ F1 plants, as we have studied, grow slightly faster. Nevertheless, although IDS RZ F1 plants were bigger than Maduro, both had the same number of leaves, meaning that IDS RZ F1 has a more open growth, which makes harvesting, and general handling of the crop, easier.

Taking together that inoculation with entomopathogenic fungi has been shown to protect plants against pests and pathogens (Bamisile et al., 2018; Vega, 2018) and that our results clearly show that inoculation of sweet pepper with entomopathogenic fungi enhances plant growth, these fungi have the potential for multitarget effects in crops on both growth promotion and biocontrol. However, the underlying mechanisms remain to be unraveled. Enhanced plant growth might have been facilitated via improved acquisition of nutrients, phytohormone production, induced resistance, production of antibiotics and secondary metabolites, and/or production of siderophores (Vega, 2018; Baron and Rigobelo, 2022). For example, inoculation of potato with *Metarhizium brunneum* resulted in an increased leaf area and plant weight, which was correlated with an increased amount of nitrogen and phosphorous content, and an increased water use efficiency (Krell et al., 2018). Which scenario is at play for the fungi investigated in our study, remains to be unraveled. Further, more research is required on the secondary metabolites produced by these endophytic entomopathogenic fungi, which may possibly end up in the fruits, as some have been found to possibly be toxic to mammals (including humans), such as beauvericin (Hu et al., 2016; Mallebrera et al., 2018).

In conclusion, our results indicate that plant root inoculation with entomopathogenic fungi enhanced overall plant growth of sweet pepper, but effects depend on fungal strain and crop cultivar. Effects also differed between years, suggesting that environmental factors can influence the outcome of endophytic colonization by entomopathogenic fungi on plant growth. Strongest plant growth promoting effects were observed for cv IDS RZ F1 inoculated with *C. fumosorosea* ARSEF 3682, expressed by enhanced canopy area and increased plant weight. These results open possibilities for the implementation of plant inoculation with entomopathogenic fungi as plant growth promoters to support and stimulate sustainable agriculture.

## Data availability statement

The original contributions presented in the study are included in the article/**Supplementary Material**. Further inquiries can be directed to the corresponding author.

## Author contributions

LW, HJ and BL designed the experiment, discussed the data, and revised the manuscript. LW and NRP performed the experiment. LW analyzed data, and prepared the first draft. All authors contributed to the article and approved the submitted version.
