# Supplementary material for: Fungal strain and crop cultivar affect growth of sweet pepper plants after root inoculation with entomopathogenic fungi
Source: Front Plant Sci. 2023 Jun 5;14:1196765. doi: 10.3389/fpls.2023.1196765 (PMC10277683; doi:10.3389/fpls.2023.1196765)
Supplement: Supplementary file 1 [file DataSheet_1.pdf]

Supplementary Material

Fungal strain and crop cultivar affect growth of sweet pepper plants after root inoculation with entomopathogenic fungi

Liesbet Wilberts, Nicolas Rojas Preciado, Hans Jacquemyn and Bart Lievens*

*** Correspondence:** Corresponding author: bart.lievens@kuleuven.be

1 Supplementary Figures and Tables

1.1 Supplementary Figures

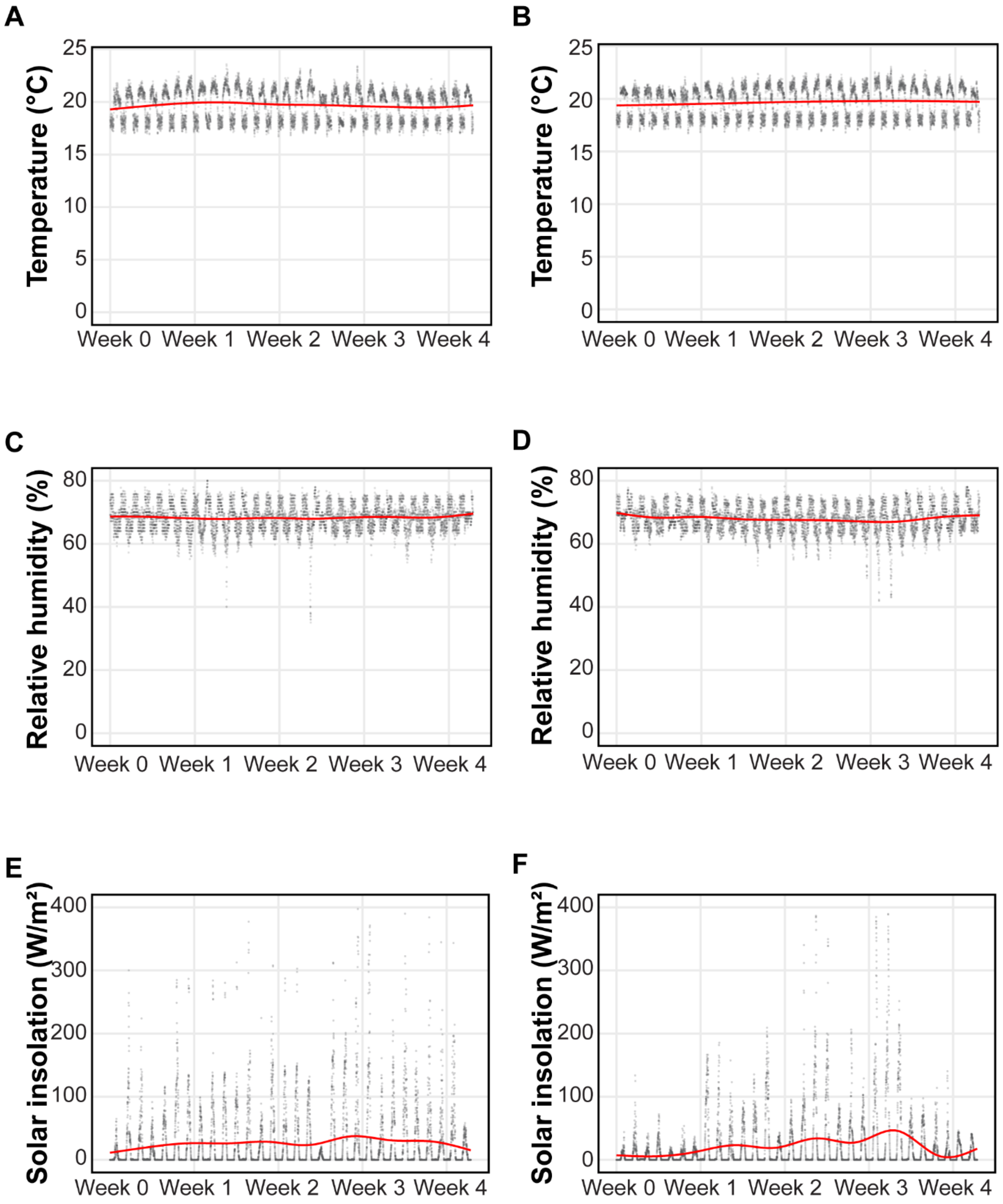

Figure S1. Measured temperature (A, B), relative humidity (C, D) and solar insolation (E, F) inside the greenhouse compartment during the experiments performed in 2021 (A, C, E) and 2022 (B, D, F). Each data point represents a measurement every 5 minutes, while the red line represents the daily mean.

1.2 Supplementary Tables

Table S1. Chemical analysis of the potting mixture used in the experiments[1].

General parameters	
pH	6.09
EC (at 25°C)	453.5 µS/cm

Inorganic composition	
B^{3+}	0.30 mg/L
Cl^-	39.34 mg/L
Extractable Ca	2684.88 mg/L
Extractable Cu	0.01 mg/L
Extractable Fe	2.89 mg/L
Extractable K	467.69 mg/L
Extractable Mg	528.07 mg/L
Extractable Mn	15.24 mg/L
Extractable Na	26.20 mg/L
Extractable P	134.65 mg/L
Extractable Zn	1.92 mg/L
NO_3^--N + NO_2-N	43.32 mg N/L
NH_4^+-N	22.84 mg N/L
SO_4^{2-}	460.74 mg/L

[1]A sample of 1L was taken from the 3:1 mixture of potting mix (Universal potting mix; Agrofino, Ghent, Belgium) and white sand. The analysis was performed by the Soil Science Institute Belgium (Bodemkundige Dienst van België; Leuven, Belgium).

Table S2. Composition of nutrient solution[1].

Nutrients	
$CA(NO_3)_2.4H_2O$	11.51 g/L
KNO_3	2.46 g/L
EDTA	0.19 mL/L
K_2SO_4	2.55 g/L
$MgSO_4.7H_2O$	3.60 g/L
KH_2PO_4	1.99 g/L
$(NH_4)_6Mo_7O_{24} \cdot 4H2O$	0.86 mg/L
$ZnSO_4(7H_2O)$	4.21 mg/L
$CuSO_4(5H_20)$	1.22 mg/l
$MnSO_4(H_2O)$	57.68 mg/L
$Na_2B_4O_7.10H_2O$	23.98 mg/L

[1]Nutrient concentration per liter, in demineralized water. Studied plants were watered daily with the nutrient solution throughout the entire experiment of four weeks. The solution was freshly made every week.
